# A comparison of population viability measures

**DOI:** 10.1002/ece3.9752

**Published:** 2023-01-24

**Authors:** Mario Trouillier, Katrin M. Meyer, Luca Santini, Guy Pe'er

**Affiliations:** ^1^ Institute for Botany and Landscape Ecology University Greifswald Greifswald Germany; ^2^ Department of Ecosystem Modelling University of Göttingen Göttingen Germany; ^3^ Department of Biology and Biotechnologies "Charles Darwin" Sapienza Università di Roma Rome Italy; ^4^ Department of Ecosystem Services UFZ ‐ Helmholtz Centre for Environmental Research Leipzig Germany; ^5^ German Centre for Integrative Biodiversity Research (iDiv) Halle‐Jena‐Leipzig Leipzig Germany

**Keywords:** extinction, population dynamics, population‐viability analysis, PVA, survival

## Abstract

The viability of populations can be quantified with several measures, such as the probability of extinction, the mean time to extinction, or the population size. While conservation management decisions can be based on these measures, it has not yet been explored systematically if different viability measures rank species and scenarios similarly and if one viability measure can be converted into another to compare studies. To address this challenge, we conducted a quantitative comparison of eight viability measures based on the simulated population dynamics of more than 4500 virtual species. We compared (a) the ranking of scenarios based on different viability measures, (b) assessed direct correlations between the measures, and (c) explored if parameters in the simulation models can alter the relationship between pairs of viability measures. We found that viability measures ranked species similarly. Despite this, direct correlations between the different measures were often weak and could not be generalized. This can be explained by the loss of information due to the aggregation of raw data into a single number, the effect of model parameters on the relationship between viability measures, and because distributions, such as the probability of extinction over time, cannot be ranked objectively. Similar scenario rankings by different viability measures show that the choice of the viability metric does in many cases not alter which population is regarded more viable or which management option is the best. However, the more two scenarios or populations differ, the more likely it becomes that different measures produce different rankings. We thus recommend that PVA studies publish raw simulation data, which not only describes all risks and opportunities to the reader but also facilitates meta‐analyses of PVA studies.

## Graphical abstract

Population viability was ranked similarly in a quantitative comparison of eight measures of population viability derived from simulated population dynamics of more than 4500 virtual species. However, direct correlations between measures were weak and could not be generalized. We thus recommend that population‐viability analyses publish raw simulation data to support robust comparisons of population viabilities.

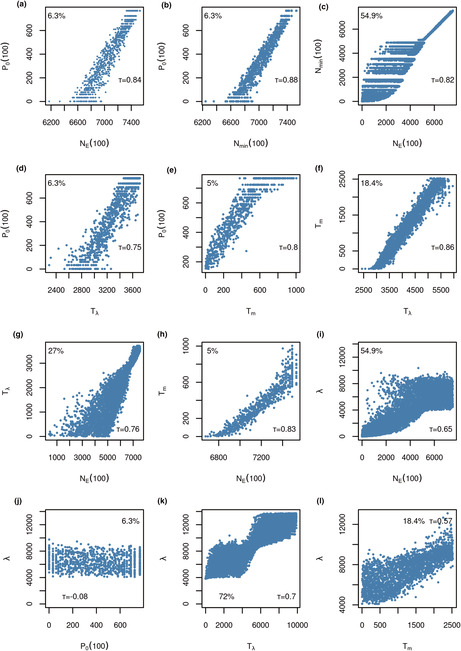

## 1 INTRODUCTION

Population‐viability analyses (PVAs) are broadly used in ecology to assess the potential development of populations over time, to characterize their current status and future development, and to suggest effective conservation interventions (Beissinger & McCullough, 2002). Even though PVAs have been criticized for being too imprecise or are of low quality (Chaudhary & Oli, 2020, 2021; Morrison et al., 2016), they are still considered a helpful tool in conservation biology (Brook, 2000; Brook et al., 2002), in particular, to evaluate the status, threats, and management options for populations (Lacy, 2019). Soulé (1987) defined viability as the minimum conditions for long‐term persistence and adaptation of populations, following the concept of the minimum viable population (Shaffer, 1981). According to Soulé (1987), population viability involves a range of properties beyond persistence, including genetic properties, individual vigor, fertility, and fecundity. Various measures exist to quantify population viability, among them the mean time to extinction and the probability of extinction. Multiple attempts have been made to improve existing viability measures or to introduce new measures, e.g., the expected minimum population size *N*_min_ (*t*) (McCarthy & Thompson, 2001) or the intrinsic mean time to extinction *T*_m_ (Grimm & Wissel, 2004). However, no measure has been proposed, or broadly adopted, that can successfully be applied to a broad range of questions of conservation practitioners or to compare different viability studies. The lack of a unifying measure probably results from the complexity of the viability concept and reflects the multifaceted nature of extinction risk, as well as the diversity of questions that PVA is used to answer.

Viability measures can be roughly categorized into three classes, namely probabilistic measures, time measures, and population‐size measures. (1) Probabilistic measures, especially the probability of extinction *P*_0_ (*t*), were the earliest and most widely used class of viability measures. Probabilities of extinction, quasi‐extinction (Ginzburg et al., 1982), or the risk of decline focus on the likelihood of extinction or falling below critical population‐size thresholds within a defined time horizon. Therefore, they require setting population size (*N*) and time thresholds (*t*), thus incorporating a subjective decision into viability assessment. (2) Time measures, especially the mean time to extinction, are frequently used as well (Foley, 1994; Reed et al., 2002). They highlight the temporal component of viability and the crucial role of population survival. The importance of time measures was underlined by the development of the intrinsic mean time to extinction *T*_m_ (Grimm & Wissel, 2004), which considers the skewness of the distribution of extinction times (Ludwig, 1996) and the probability of reaching the established phase. (3) Examples of population‐size measures are the expected average population size *N*_E_ (*t*) and the expected minimum population size *N*_min_ (*t*) (McCarthy & Thompson, 2001). In addition to these three rough categories, the population growth rate *λ* is not a traditional viability measure but a very important population property which is clearly related to viability, as it describes the trend of a population size (declining, stable, or increasing) (Lande, 1993). We therefore regard it here as a viability measure as well.

Together, over 20 different viability measures have been used in the literature (Pe'er et al., 2013), demonstrating the multidimensionality of the viability concept. It has been argued (e.g., Burgman et al. (1993)) that different viability measures might address different questions. For instance, the probability of extinction can help decision‐makers assess how necessary it is to act, while the mean time to extinction might be suitable to assess how urgent an intervention may be. Choosing the best of several conservation actions could be done based on the expected population size at a given time (e.g., 10 years) after an intervention was taken.

Attempts to compare the results from multiple PVA studies that used different viability measures concluded that quantitative comparisons and generalizations remain virtually impossible, and little progress has been made over time (Burgman & Possingham, 2000; Crone et al., 2011; Naujokaitis‐Lewis et al., 2009; Pe'er et al., 2013; Shaffer et al., 2002). There remains a need to assess different measures in terms of their consistency and suitability for different purposes. Ideally, such an assessment could guide the choice of viability measures and help mitigating the risk that the choice of a certain measure over another may affect the outcomes (e.g., in terms of the proposed intervention). Furthermore, it would be useful to identify the quantitative relationships between viability measures, in order to advance potential attempts for integration and quantitative analyses across studies to foster generalizations. If different viability measures ranked the same set of populations, species, or scenarios differently, this would complicate decision‐making in nature conservation. By contrast, a consistent ranking of viability measures would enhance comparability.

This study compares eight viability measures: probability of extinction *P*_0_ (*t*), risk of decline to a threshold population size *P*_N_ (*t*), probability of quasi‐extinction *P* *_Q_*_*E*,*N*_ (*t*) (Ginzburg et al., 1982), mean expected population size *N*_E_ (*t*), expected minimum population size *N*_min_ (*t*) (McCarthy & Thompson, 2001), expected/mean time to extinction *T*_E_, intrinsic mean time to extinction *T*_m_ (Grimm & Wissel, 2004), and population growth rate *λ*. These eight viability measures were chosen because they are commonly used in the literature or proposed to be key measures for extracting important information from PVA simulations (Grimm & Wissel, 2004; IUCN, 2012). We put emphasis on measures that represent the classes mentioned above, namely probabilistic, time, and population‐size measures, as well as the growth rate as a measure to characterize populations' trajectory over time.

To evaluate the differences between these measures we simulated virtual species with diverse life histories on different habitat maps. From the model output, we computed the eight viability measures and tested: (a) if different viability measures ranked species and scenarios differently, (b) if viability measures correlate and if one measure can directly be computed from another, and (c) if the simulation model and scenario parameters affect the relationship between two viability measures.

## 2 MATERIALS AND METHODS

### 2.1 Simulating virtual species

Viability measures are computed from modeled population‐size time series. Thus, we first parametrized the agent‐based model RangeShifter (Bocedi et al., 2014) to simulate populations. The model allows a detailed parameterization that fits the life histories of a wide variety of species. For the parameterization, we used a published dataset that covers the parameters of 4574 virtual mammals. This dataset was created to cover the diversity of sizes and life histories of real animals while accounting for the collinearity of different characteristics (Santini et al., 2016). The simulated species vary with respect to body mass, sexual maturity age, litters per year, litter size, home range area, population density, dispersal distance, and annual survival rate. All species were simulated with 100 repetitions for 100 years on three artificial fractal habitat maps. The habitat maps were created with RangeShifter (65 × 65 cells, Hurst exponent = 0.1) with 5%, 10%, and 20% habitat cover to reflect landscapes of different suitability to the species. This resulted in 13,722 scenarios (3 maps × 4574 species). Map resolution and extent were adapted to account for the large differences in species size and life histories. The RangeShifter model returned 100 time series of population sizes over 100 years for each species. These time series were then used to calculate the viability measures.

To assess if the parameters of the simulation model can affect the relationship between two viability measures, we created three additional sets of scenarios: In each set, we varied either the carrying capacity of habitat patches, the mean dispersal distance, or the fraction of habitat patches in the map, while not changing any of the other parameters.

All RangeShifter parametrization files and outputs can be found in the Appendix S1.

### 2.2 Computing viability measures

For each simulated scenario, we calculated eight viability measures in the following way:

1. Probability of extinction *P*_0_ (*t*): the share of simulation runs in which an extinction (population size = 0) occurred within the specified time horizon *t*.
2. Risk of decline *P*_N_ (*t*): the proportion of simulation runs in which the population size was equal to or lower than a population‐size threshold *N* after the specified time horizon *t*.
3. Probability of quasi‐extinction *P*_QE,N_ (*t*) (Ginzburg et al., 1982): the fraction of simulation runs in which the population size dropped at least once below a population‐size threshold *N* within the specified time horizon *t*.
4. Expected population size *N*_E_ (*t*), also referred to as the mean population size, was calculated as the average population size of all simulation runs at time *t*.
5. Expected minimum population size *N*_min_ (*t*) was obtained by calculating the mean of every simulation run's minimum population size within the time horizon *t* (related to the concept of the minimum viable population (Gilpin & Soulé, 1986)).
6. Intrinsic mean time to extinction *T*_m_ was calculated from the probability of extinction over time, as the inverse slope of the linear regression through the tail of the **–**ln (1 *− P*_0_) graph (Grimm & Wissel, 2004).
7. The mean time to extinction *T*_E_ was extrapolated from the mean population size and the growth rate *λ* (intercept at *N*_E_ (*t*) = 0). This allowed to compute *T*_E_ even when not all simulation runs led to extinction within the simulated time frame.
8. The growth rate *λ* was calculated as the slope of the linear regression line of the mean population‐size time series.

The viability measures 1–5 require further specifications of a time horizon (*t*) and/or a population‐size (*N*) threshold. We used 25, 50, 75, and 100 years as time horizons and population‐size thresholds of 1%, 5%, and 10% of the initial population size.

### 2.3 Comparing viability measures

In this study, we (a) compared scenario rankings to find out if viability measures ranked scenarios the same, (b) explored if viability measures correlated directly or whether it was possible to calculate one measure from another, and (c) evaluated if the relationship between any two viability measures was affected by the parameters in the simulation model.

First, we evaluated if the rankings produced by the different measures were consistent with each other. To this end, we ranked all species based on each of the eight viability measures and used Kendall rank correlation coefficients to compare if different measures resulted in a similar ranking. Ties were handled by assigning the same rank and skipping one level (e.g., two species with rank 1 were followed by a rank of 3).

Second, we explored direct correlations and mathematical relationships between different viability measures that might allow for converting one measure into another. To do so, we fitted various linear and nonlinear models using the *nls2* package (Baty et al., 2015; R Core Team, 2015) in R (R Core Team, 2015).

Lastly, for a more detailed assessment of the relationship between two viability measures, we explored if changes in single model parameters altered these relationships. In particular, we changed the carrying capacity per habitat patch and the mean dispersal distance (negative exponential dispersal kernel), and we used different habitat maps with different fractions of habitat (Appendix S2). If single model parameters caused changes in the relationship between viability measures, it would indicate that fixed functional relationships between viability measures might not exist. We thus computed the probability of extinction and the expected population size for all 100 years and plotted these values against each other for each parameter setting.

## 3 RESULTS

### 3.1 Viability rankings

**FIGURE 1:**
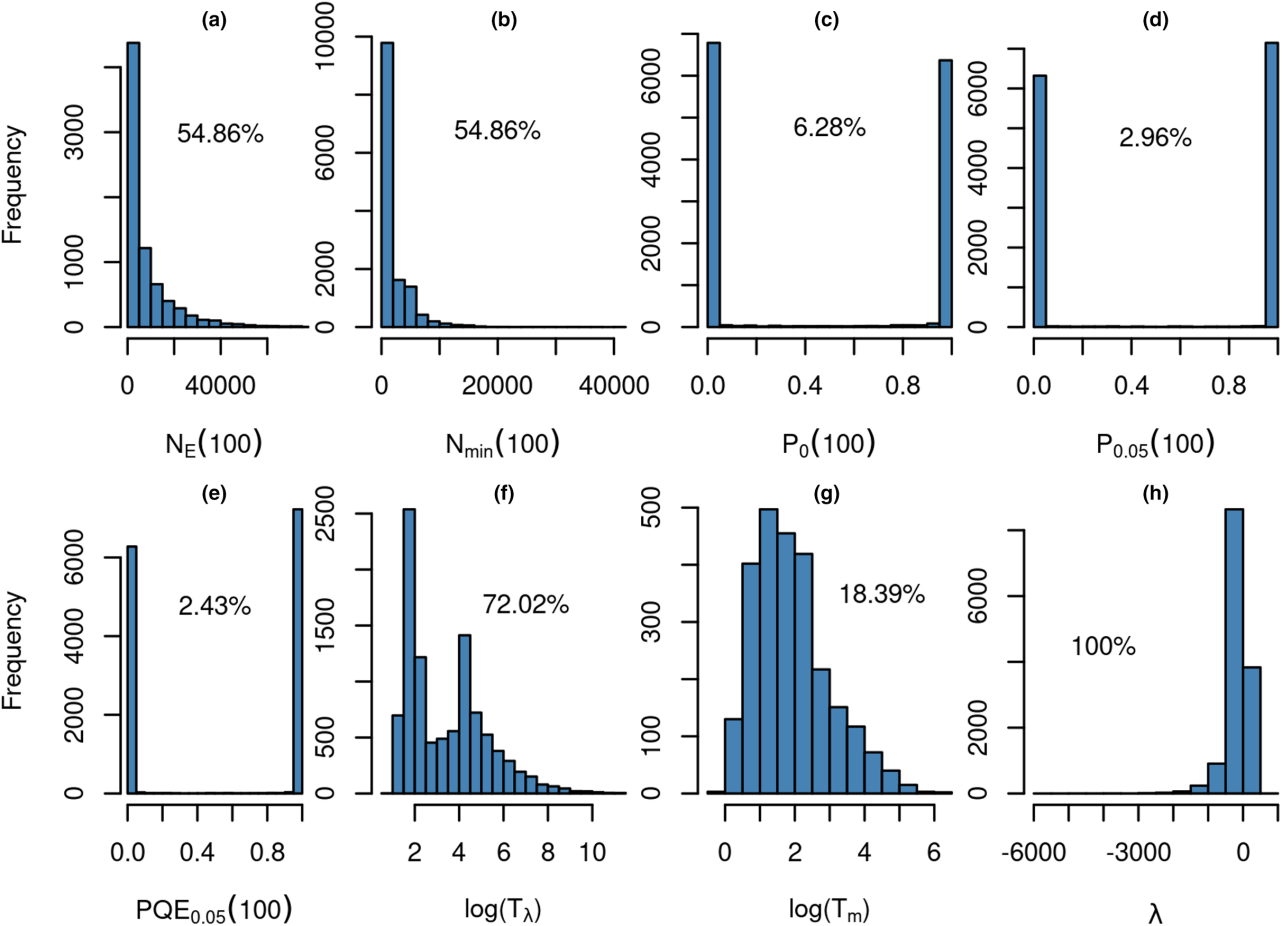
Frequency distributions of the eight viability measures that were computed for the 13,722 scenarios. Percentage values indicate the fraction of scenarios for which each measure could be calculated and returned meaningful values.

The computed viability measures show that each measure only worked for a fraction of all scenarios (Figure 1). For example, the population‐size measures *N*_E_ (100) and *N*_min_ (100) returned 0 for more than 50% of all scenarios. In those scenarios, the populations always went extinct before 100 years. Similarly, the probability of extinction after 100 years, *P*_0_ (100), returned either 0 or 1 for 93.6% of all scenarios. For the same reason, the mean (extrapolated) time to extinction could only be calculated for 77.65% of all scenarios because the remaining scenarios were stable or showed a positive growth trend.

**FIGURE 2:**
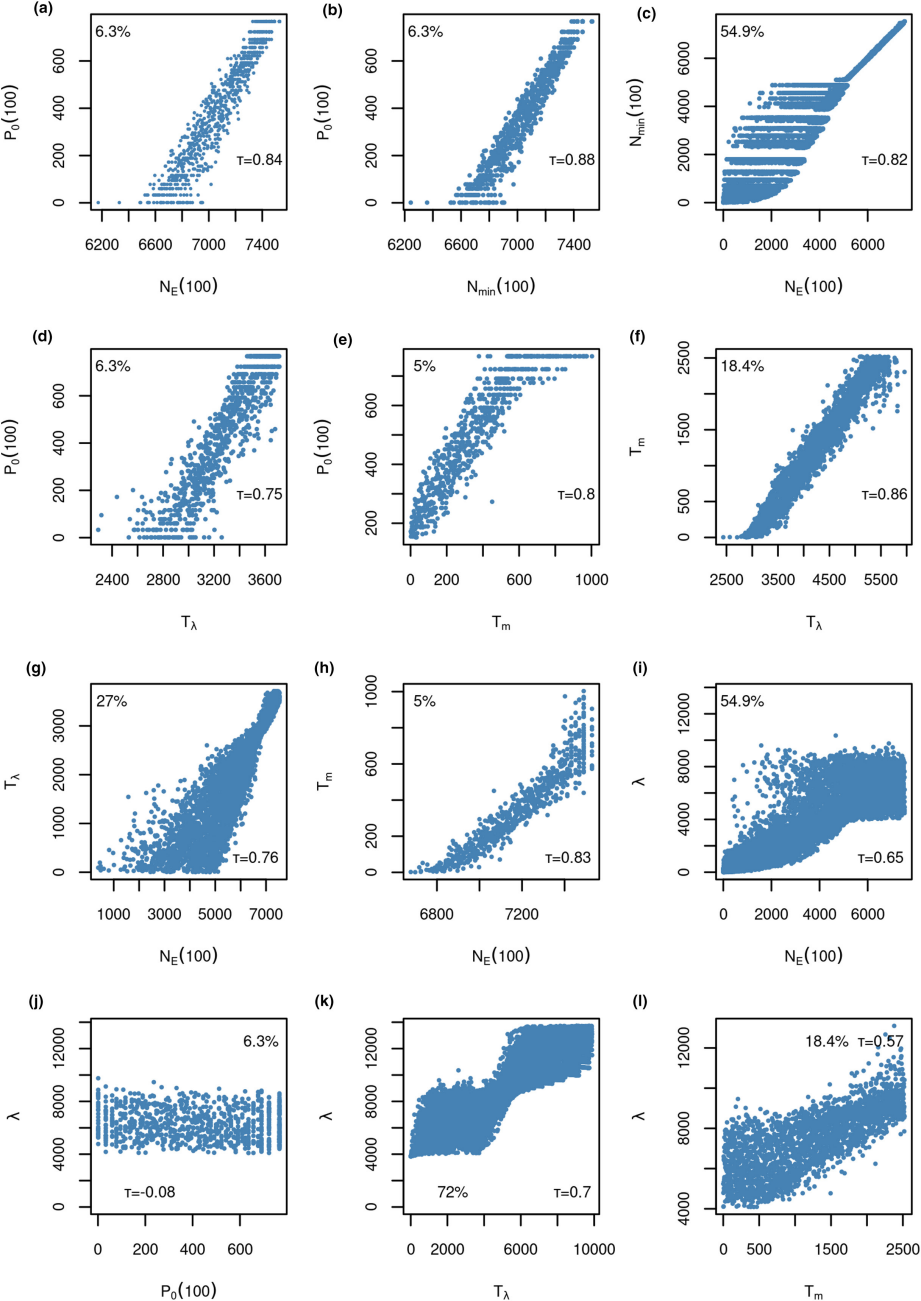
Relationships between different viability measures' scenario rankings. Percentage values indicate the fraction of scenarios where both measures returned meaningful values. Additionally, Kendall's correlation coefficient (*τ*) is shown. We selected a broad range of all possible pairwise combinations of measures representative of all possible relationships.

Pairwise correlations of viability measures showed positive correlations of varying strength (Figure 2). Kendall rank correlation coefficients were only computed for those scenarios where the pairs of viability measures both returned meaningful values. Correlation coefficients ranged from 0.65 to 0.88, except when the growth rate was involved. Growth rate versus mean time to extinction had a correlation coefficient of 0.57 and the correlation between growth rate and probability of extinction got as low as 0.08 (Figure 2). Most species and scenarios were ranked relatively similarly by the different viability measures, and the relationship was often mostly linear but rarely was the ranking exactly the same for two measures. The growth rate *λ* was a notable exception to this trend because its rankings differed greatly from all other rankings (Figure 2).

### 3.2 Functional relationships between viability measures

**FIGURE 3:**
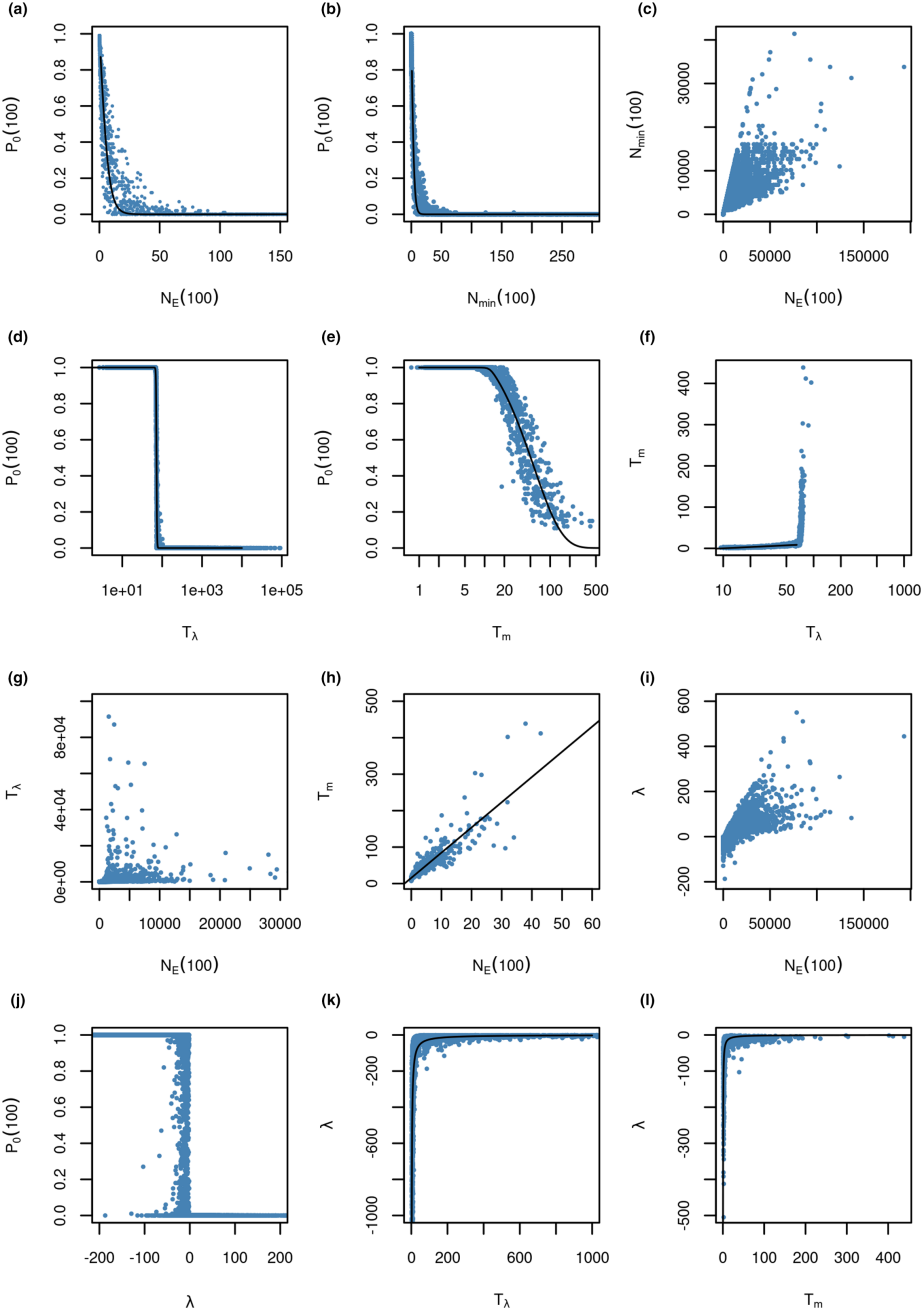
Relationships between different viability measures. For some measures, the functional relationship can roughly be described with adapted reciprocal functions (e.g., a, b, k, l), logistic functions (e.g., d, e), or simple linear functions (e.g., h), as shown in Table 1. However, significant variance and heteroscedasticity rendered even these relationships mostly useless to reliably calculate one measure from another.

The quantitative relationship between viability measures was in some cases linear but more often nonlinear (Figure 3, Table 1). Often, these functions describe asymptotes, for example, the probability of extinction approaches zero at high population sizes (Figure 3a). This is related to the same issue described above, that certain viability measures only returned meaningful values within a limited viability range. Additionally, various issues impeded the conversion of one viability measure into another. For example, the variance between *N*_E_ and *N*_min_ (Figure 3c) increased greatly toward larger values. Another example was the relationship between *T*_m_ and *T*_E_, which showed a breakpoint (Figure 3f), which is an artifact related to the length of the modeled time period (100 years), while the relationship before this breakpoint was approximately linear (Table 1). A positive growth rate always corresponded to a *P*_0_ (100) of zero. On the other side, even strongly negative growth rates were sometimes linked to a *P*_0_ (100) of zero, if the population size was very large. Lastly, some measures showed a distinct relationship when one or both measures were log‐transformed (e.g., Figure 3e,f). On a log–log scale even very coarse correlations can look meaningful, but in practice, this will hardly be useful to compute one measure from another because it hides the large variance (e.g., *N*_E_ vs. *T*_E_).

**TABLE 1:** Approximated functional relationships between selected viability measures as shown in Figure 3

Viability measure 1	Viability measure 2	Approximated functional relationship
*P*_0_ (100)	*N*_E_ (100)	$P_{0}\;\left(100\right)=\frac{2}{1+1.295^{N_{E}\left(100\right)}}$
*P*_0_ (100)	*N*_min_ (100)	$P_{0}\;\left(100\right)=\frac{2}{1+1.518^{N_{\min}\left(100\right)}}$
*P*_0_ (100)	*T*_E_	$\mathrm{Po}\left(100\right)=\frac{1}{1+e^{0.965\times \left(T_{E}-72.285\right)}}$
*P*_0_ (100)	*T*_m_	$P_{0}\;\left(100\right)=\frac{1}{{\left(1+e^{1.272\times \left(T_{m}-11.753\right)}\right)}^{0.014}}$
*T*_m_	*T*_E_	$T_{m}=4.521\times T_{E}-10.371$
*T*_m_	*N*_E_ (100)	$T_{m}=6.914\times N_{E}+15.622$
*λ*	*T*_E_	$\lambda =\frac{1}{-0.0004\times T_{E}}$
*λ*	*T*_m_	$\lambda =\frac{1}{-0.0062\times T_{m}}$

### 3.3 The effect of model parameters on relationships between viability measures

**FIGURE 4:**
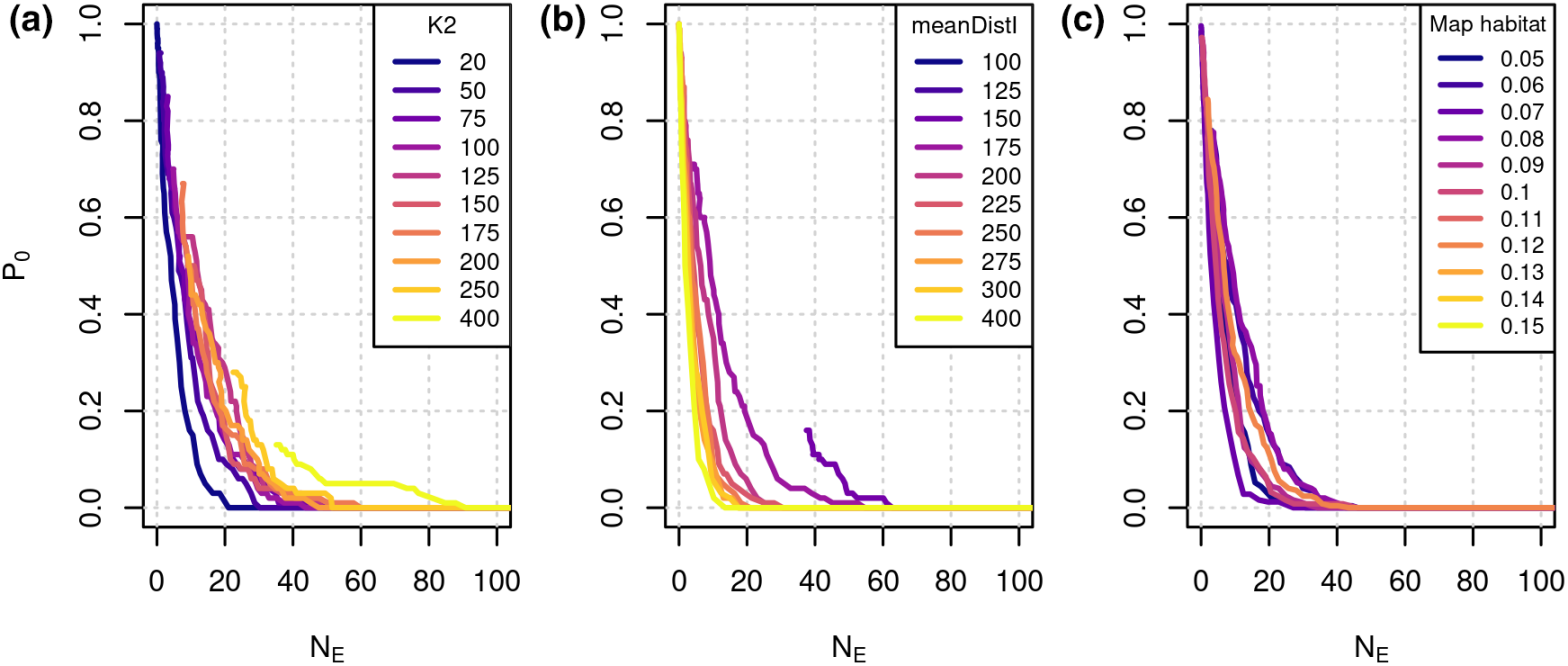
The relationship between the expected population size (*N*_E_) and the probability of extinction (*P*_0_) depends on scenario parameters. Here, all RangeShifter parameters were kept constant, except (a) the carrying capacity per habitat patch (*K2*), (b) the mean dispersal distance (*meanDistI*), and (c) the map with different fractions of suitable habitats in the landscape (scenarios that are not plotted showed no extinctions).

We found that changing parameters in the simulation model altered the relationship between viability measures. In particular, changing the carrying capacity, mean dispersal distance, or the habitat map altered the relationship between *P*_0_ and *N*_E_ (Figure 4). For example, at a given *P*_0_, *N*_E_ increased with increasing carrying capacity (Figure 4a) and with decreasing mean dispersal distance (Figure 4b). This dependence was slightly weaker when considering the change in *P*_0_ at a given *N*_E_ (Figure 4a–c). We also note that there were threshold behaviors, such as a decrease in the maximum possible P0 with decreasing mean dispersal distances (Figure 4b) and a complete absence of extinctions when the proportion of suitable habitat exceeded about 10% (Figure 4c). This means that the same population size can correspond to different probabilities of extinction, which likely also partly explains the low correlation strength between different viability measures (Figure 3). Consequently, we did not find any universal relationship between viability measures, that would not be sensitive to simulation model parameters.

## 4 DISCUSSION

Our systematic comparison of eight different population‐viability measures across different scenarios and species showed three main results: First, all viability measures, except the growth rate *λ*, ranked the population viability of the simulated species similarly but not identically. Second, we found rough correlations, but no fixed relationships between viability measures, that would allow the conversion of one measure into another. Third, species and scenario parameters of the simulation model (including the habitat map) altered the relationship between any two viability measures. Consequently, it appears to be impossible to compute one viability measure directly from another one. At best, functional relationships between two measures could be approximated for very similar scenarios. Hereafter, we outline the causes and implications of these findings and discuss whether a single number can represent viability.

### 4.1 The relationships between viability measures

Our result that different viability measures rank species or scenarios similarly and that at least some viability measures correlate, indicates that most measures are based on a similar concept of viability. As a result, identifying the best management option for a population seems to be robust with respect to the choice of the viability measure. By contrast, some scenario rankings were not identical and it was not possible to determine fixed relationships between viability measures. Thus, there are cases where the choice of the viability measure will affect which management option is considered the best for a population or which population is deemed more viable. Furthermore, our results imply that two studies that reported two different viability measures cannot directly be compared by converting one measure into the other.

The relationships between viability measures seem to depend on species traits, carrying capacity, and habitat configuration. For example, increasing the species trait dispersal distance reduced the population size N_E_ at a given extinction probability *P*_0_. This may be due to more intra‐ and interspecific interactions when species cover greater distances. This explanation is in line with our observation of decreasing maximum possible values of *P*_0_ with decreasing dispersal distances. Furthermore, carrying capacity and the proportion of suitable habitat modified the relationship between *N*_E_ and *P*_0_ in an intuitive way, i.e., *N*_E_ increased with increasing carrying capacity and *P*_0_ became zero beyond a 10% threshold of habitat suitability. These are interesting theoretical interdependencies, but conservation scientists may often not have enough species trait and habitat data to assess these dependencies in detail. Thus, a pragmatic recommendation for conservation scientists, especially when supporting on‐the‐ground measures for population management, would be to choose (several) viability measures that show the least dependence on traits. In our case, *P*_0_ should, for example, be chosen over N_E_ because it was relatively less affected by differences in the traits we investigated (Figure 4). However, such dependencies may not always be as straightforward nor as intuitive as in our study. Moreover, relationships of viability measures may respond differently to distinct trait syndromes, such as fast versus slow pace‐of‐life syndromes (Healy et al., 2019), and these dependencies may be subject to spatial or temporal variability. Taken together, this calls for more research into the trait dependence of viability measures and the relationships between viability measures.

**FIGURE 5:**
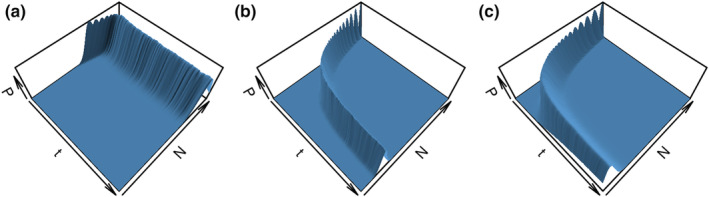
Three examples of probability distributions (*P*) of population sizes (*N*) over time *t*. The distributions show (a) a population that stabilizes very early at a high level and also shows a high variance, (b) a population whose size first decreases but then stabilizes with a low variance at a certain population size, and (c) a population that declines and where some simulation runs already led to extinction.

The lack of fixed relationships between viability measures can be explained by how viability measures process raw data. First, many viability measures are only based on a data subset, for example, *N*_E_ (100) only requires the population size of all scenario repetitions after 100 years but discards all other information. Similarly, *P*_0_ (100) only evaluates the fraction of simulation runs that went extinct after 100 years. Second, each viability measure aggregates the data in a unique way into a single number. Of course, the goal of a viability measure is exactly this, to describe viability in a single number, but this necessarily entails a loss of information regarding the underlying data distribution. A useful analogy is the computation of mean and median: Both can be calculated for the same distributions and both values will correlate when computed for a number of datasets. However, it is arguably not very meaningful to compute the mean from the median and vice versa. The same effect applies to viability measures. Each modeled scenario will result in a unique population‐size frequency distribution over time (Figure 5). These 3D distributions are characterized by different means, skewness, kurtosis, and how these characteristics change over time. Viability measures intend to summarize all the information from these distributions into a single number, but from this number, one cannot reconstruct the original distribution. Consequently, one cannot accurately calculate one viability measure from another.

### 4.2 Can a single number describe viability?

Given that there are many ways to aggregate raw population‐viability data into a single number and that they all entail an information loss (Table 2), it seems questionable if viability can or should be expressed as a single number. However, if viability is not expressed as a single number, it is also not possible to objectively rank different scenarios because only single numbers, not distributions, can be ranked at all. Thus, if we want to rank scenarios to support management decisions, what would be the most suitable single number viability measure (acknowledging that none of them will be perfect)?

**TABLE 2:** Advantages and disadvantages of the analyzed viability measures.

Measure	Advantages	Disadvantages
*Probabilistic measures*		
*P*_0_ (*t*)—probability of extinction	focuses on population survival	extinctions need to happen within the modeled time horizon requires defining a time horizon only returns meaningful values (0 < *P*_0_ (*t*) < 1) for a fraction of all scenarios
*P*_N_ (*t*)—risk of decline	incorporates that small population sizes are almost certainly doomed (extinction vortex (Gilpin & Soulé, 1986))	requires defining a time horizon requires population‐size threshold only returns meaningful values (0 < *P*_N_ (*t*) < 1) for a fraction of all scenarios
*P*_QE,N_ (*t*)—probability of quasi‐extinction	gives even more weight to the extinction vortex than the risk of decline	requires defining a time horizon requires population‐size threshold only returns meaningful values (0 < *P*_QE,N_ (*t*) < 1) for a fraction of all scenarios
*Time measures*		
*T*_m_—intrinsic mean time to extinction	aggregates population sizes and growth rate considers skewness in the extinction‐time distribution (Grimm & Wissel, 2004)	extinctions need to happen within the modeled time horizon if the probability of reaching the established phase is <1 viability rankings are not possible
*T*_e_ *—*(extrapolated) mean time to extinction	aggregates population sizes and growth rate	does not consider skewness in the extinction‐time distribution
*Population‐size measures*		
*N*_E_ (*t*)—expected population size	focuses on the state of a population	requires defining a time horizon neglects growth rate
*N*_min_ (*t*)—expected minimum population size	changes more gradually than the risk of decline (McCarthy & Thompson, 2001) considers extinction vortices	requires time horizon requires population‐size threshold neglects growth rate
λ—growth rate	focuses on the change in population size	must be interpreted in combination with population size only stable if model parameters do not change over time

Extinction and survival are at the core of the viability concept. At first sight, this might imply that population sizes or growth rates are nonideal proxies of viability, because, by definition, a single surviving individual is sufficient to prevent the extinction of a species. However, population sizes and growth rates do affect viability via their effect on the occurrence and timing of extinctions. Nevertheless, measures related to population size or growth rate capture viability less explicitly than measures related to extinction probability. Thus, the extinction probability distribution over time, *P*_0_ (*t*), is the most fundamental description of viability.

To rank scenarios by *P*_0_ (*t*) requires to aggregate a distribution into a single number. The probability of extinction at one (more or less arbitrary) point in time, e.g., 100 years, is one way to summarize the *P*_0_ (*t*) distribution. Time measures like *T*_E_ and *T*_m_ are another way to summarize the *P*_0_ (*t*) distribution into a single number. But *T*_E_ has been criticized because the *P*_0_ (*t*) distribution is often right‐skewed (Grimm & Wissel, 2004; Ludwig, 1996), and *T*_m_ only works in stable environments (because if the environment changes in the simulated time period, the tail of the –ln (1 *− P*_0_) graph will not be linear, as required by *T*_m_).

**FIGURE 6:**
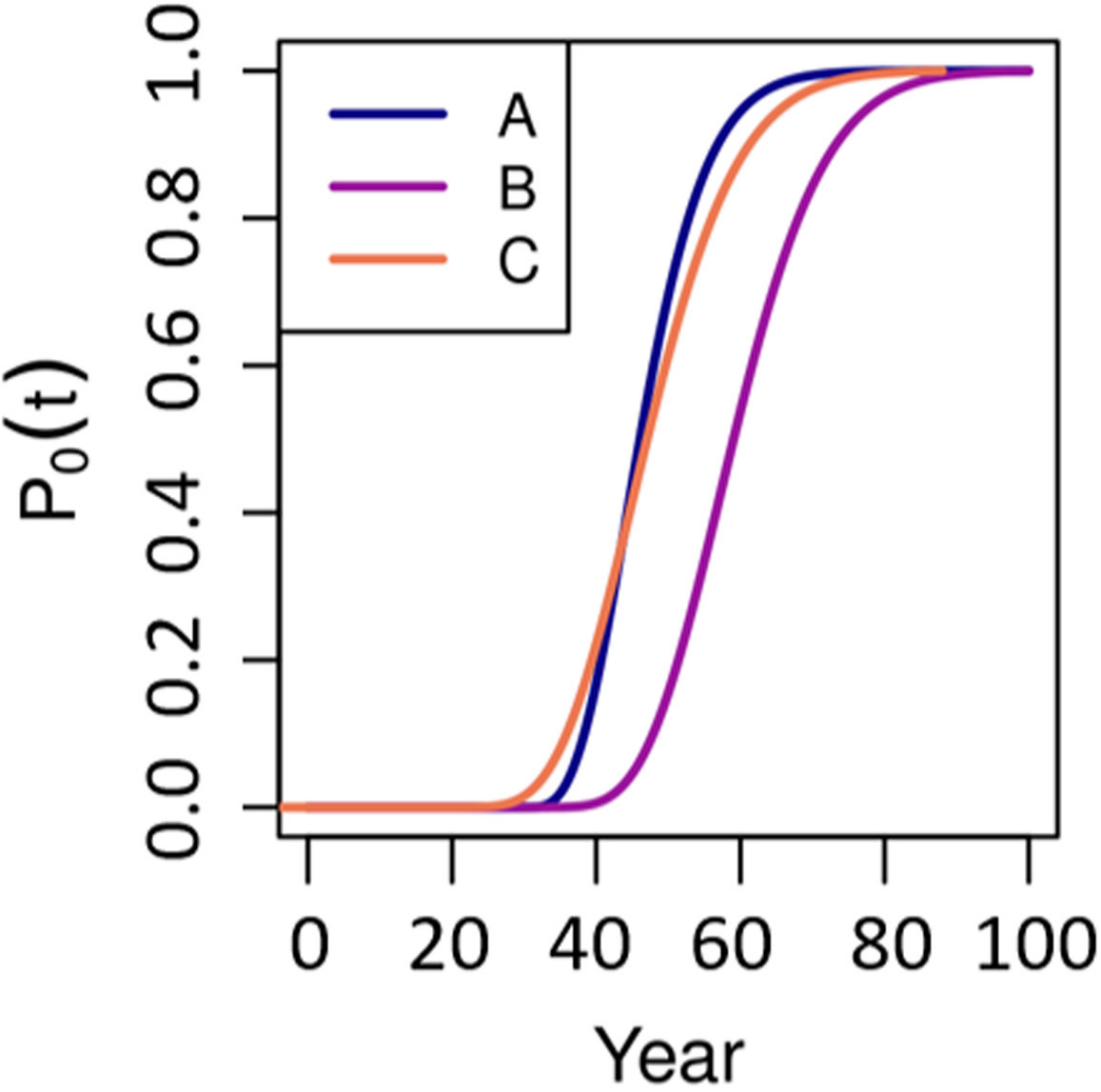
The probability of extinction distributions of three scenarios shows that ranking population viability based on distributions is not always easy. It is easy to conclude that scenarios A and C show lower viability than scenario B because the probability of extinction is higher in A and C than in B at each point in time. However, the comparison of scenarios A and C is more difficult: Scenario C offers the chance of longer survival than scenario A but also bares the risk of an earlier extinction. Arguably, these risks and chances cannot objectively be weighed against each other to conclude that one scenario is more viable than the other.

Aggregating the *P*_0_ (*t*) distribution into a single number essentially means that the risks at different time periods are weighted against each other. This weighting is subjective and depends on a person's risk affinity. For example, would you trade a 1% higher extinction risk at time *t* for a 1.1% lower extinction risk at time *t +* 1? What about a 2%, 5%, or 10% lower extinction risk at *t +* 1? While some pairs of *P*_0_ (*t*) distributions reflect clear differences in viability, distributions that, for example, mostly differ by lower or higher variance cannot be ranked objectively (Figure 6). These idiosyncrasies of *P*_0_ (*t*) distributions may be due to stochasticity effects on population dynamics (Melbourne & Hastings, 2008), as well as the typically right‐skewed extinction‐time distributions.

While measures like the *P*_0_ (100) or the (intrinsic) mean time to extinction can be seen as established conventions on how to summarize the *P*_0_ (*t*) distribution into a single number, the inherent subjectivity of this process poses a problem to any viability ranking and to any comparisons of populations, species or scenarios. Conservation scientists who support population management need to be clear about how a viability measure deals with probabilities, risks, and chances. This further supports that conservation scientists should assemble and report the raw simulated population‐size time series to facilitate the comparison of different studies because population‐size time series are the basis for computing the *P*_0_ (*t*) distribution or any other required viability measure.

## 5 CONCLUSIONS

In this study, we show that viability measures rank species or scenarios similarly but not identically. To rank species or scenarios, viability measures have to aggregate the raw population‐size time series into single numbers. This aggregation cannot be objective because it depends on how future risks and chances are weighted against each other. Furthermore, viability measures cannot be converted into each other because the specific parameterization of the population model affects the relationships between any two viability measures. Current viability measures, which have different advantages and disadvantages, represent established and useful conventions on how to quantify population viability into single numbers. For the future, however, it is advisable that PVA studies publish raw simulated population‐size time series because they have many benefits not only for theory but also for conservation practice: First and foremost, raw population‐size time series are the basis of a thorough probabilistic analysis including the possibility to determine all viability measures presented here; second, they make all risks and chances of the different analyses transparent, and finally, they allow for comprehensive and valid comparisons between studies, facilitating meta‐analyses of studies that assess population viability.

## AUTHOR CONTRIBUTIONS

**Mario Trouillier:** Conceptualization (equal); formal analysis (equal); investigation (equal); methodology (equal); software (equal); visualization (equal); writing – original draft (equal); writing – review and editing (equal). **Katrin M. Meyer:** Conceptualization (equal); investigation (equal); methodology (equal); resources (equal); supervision (equal); writing – review and editing (equal). **Luca Santini:** Data curation (equal); investigation (equal); methodology (equal); writing – review and editing (equal). **Guy Pe'er:** Conceptualization (equal); data curation (equal); investigation (equal); methodology (equal); resources (equal); software (equal); supervision (equal); writing – review and editing (equal).

## Supporting information


Appendix S1

Appendix S2

Appendix S3

## Data Availability

The data that support the findings of this study are parameters of virtual species that are available in Santini et al. (2016), Global Change Biology, at https://doi.org/10.1111/gcb.13271. The R code underlying the analysis of the data is available in the supplementary material of this article (Appendix S3).

## References

[ece39752-bib-0001] Baty, F. , Ritz, C. , Charles, S. , Brutsche, M. , Flandrois, J.‐P. , & Delignette‐Muller, M.‐L. (2015). A toolbox for nonlinear regression in R: The package nlstools. Journal of Statistical Software, 66(5), 1–21.

[ece39752-bib-0002] Beissinger, S. R. , & McCullough, D. R. (2002). Population viability analyisis. The University of Chicago Press. http://press.uchicago.edu/ucp/books/book/chicago/P/bo3637258.html

[ece39752-bib-0003] Bocedi, G. , Palmer, S. C. F. , Pe'er, G. , Heikkinen, R. K. , Matsinos, Y. G. , Watts, K. , & Travis, J. M. J. (2014). RangeShifter: A platform for modelling spatial eco‐evolutionary dynamics and species' responses to environmental changes. Methods in Ecology and Evolution, 5(4), 388–396. 10.1111/2041-210X.12162

[ece39752-bib-0004] Brook, B. W. (2000). Pessimistic and optimistic bias in population viability analysis. Conservation Biology, 14(2), 564–566.

[ece39752-bib-0005] Brook, B. W. , Burgman, M. A. , Akçakaya, H. R. , O'Grady, J. J. , & Frankham, R. (2002). Critiques of PVA ask the wrong questions: Throwing the heuristic baby out with the numerical Bath water. Conservation Biology, 16(1), 262–263. 10.1046/j.1523-1739.2002.01426.x 35701975

[ece39752-bib-0006] Burgman, M. A. , Ferson, S. , & Akçakaya, H. R. (1993). Risk assessment in conservation biology. Springer Science & Business Media.

[ece39752-bib-0007] Burgman, M. A. , & Possingham, H. P. (2000). Population viability analysis for conservation: The good, the bad and the undescribed. Cambridge University Press. https://digital.library.adelaide.edu.au/dspace/handle/2440/31127

[ece39752-bib-0008] Chaudhary, V. , & Oli, M. K. (2020). A critical appraisal of population viability analysis. Conservation Biology, 34(1), 26–40. 10.1111/cobi.13414 31435956

[ece39752-bib-0009] Chaudhary, V. , & Oli, M. K. (2021). False dichotomy in population viability analysis quality assessment: Reply to Lawson et al. Conservation Biology, 35, 1686–1688. 10.1111/cobi.13819 34405449

[ece39752-bib-0010] Crone, E. E. , Menges, E. S. , Ellis, M. M. , Bell, T. , Bierzychudek, P. , Ehrlén, J. , Kaye, T. N. , Knight, T. M. , Lesica, P. , Morris, W. F. , Oostermeijer, G. , Quintana‐Ascencio, P. F. , Stanley, A. , Ticktin, T. , Valverde, T. , & Williams, J. L. (2011). How do plant ecologists use matrix population models? Ecology Letters, 14(1), 1–8. 10.1111/j.1461-0248.2010.01540.x 21070554

[ece39752-bib-0011] Foley, P. (1994). Predicting extinction times from environmental Stochasticity and carrying capacity. Conservation Biology, 8(1), 124–137. 10.1046/j.1523-1739.1994.08010124.x

[ece39752-bib-0012] Gilpin, M. E. , & Soulé, M. E. (1986). Minimum viable populations: The processes of species extinctions. Sinauer Associates.

[ece39752-bib-0013] Ginzburg, L. R. , Slobodkin, L. B. , Johnson, K. , & Bindman, A. G. (1982). Quasiextinction probabilities as a measure of impact on population growth. Risk Analysis, 2(3), 171–181. 10.1111/j.1539-6924.1982.tb01379.x

[ece39752-bib-0014] Grimm, V. , & Wissel, C. (2004). The intrinsic mean time to extinction: A unifying approach to analysing persistence and viability of populations. Oikos, 105(3), 501–511. 10.1111/j.0030-1299.2004.12606.x

[ece39752-bib-0015] Healy, K. , Ezard, T. H. G. , Jones, O. R. , Salguero‐Gómez, R. , & Buckley, Y. M. (2019). Animal life history is shaped by the pace of life and the distribution of age‐specific mortality and reproduction. Nature Ecology & Evolution, 3, 1217–1224. 10.1038/s41559-019-0938-7 31285573

[ece39752-bib-0016] IUCN . (2012). IUCN red list categories and criteria: Version 3.1 (2nd ed.). IUCN.

[ece39752-bib-0017] Lacy, R. C. (2019). Lessons from 30 years of population viability analysis of wildlife populations. Zoo Biology, 38(1), 67–77. 10.1002/zoo.21468 30585658

[ece39752-bib-0018] Lande, R. (1993). Risks of population extinction from demographic and environmental stochasticity and random catastrophes. The American Naturalist, 142(6), 911–927. 10.1086/285580 29519140

[ece39752-bib-0019] Ludwig, D. (1996). The distribution of population survival times. The American Naturalist, 147(4), 506–526.

[ece39752-bib-0020] McCarthy, M. A. , & Thompson, C. (2001). Expected minimum population size as a measure of threat. Animal Conservation, 4(4), 351–355. 10.1017/S136794300100141X

[ece39752-bib-0021] Melbourne, B. A. , & Hastings, A. (2008). Extinction risk depends strongly on factors contributing to stochasticity. Nature, 454(7200), 100–103. 10.1038/nature06922 18596809

[ece39752-bib-0022] Morrison, C. , Wardle, C. , & Castley, J. G. (2016). Repeatability and reproducibility of population viability analysis (PVA) and the implications for threatened species management. Frontiers in Ecology and Evolution, 4, 98. 10.3389/fevo.2016.00098

[ece39752-bib-0023] Naujokaitis‐Lewis, I. R. , Curtis, J. M. R. , Arcese, P. , & Rosenfeld, J. (2009). Sensitivity analyses of spatial population viability analysis models for species at risk and habitat conservation planning. Conservation Biology, 23(1), 225–229. 10.1111/j.1523-1739.2008.01066.x 18798856

[ece39752-bib-0024] Pe'er, G. , Matsinos, Y. G. , Johst, K. , Franz, K. W. , Turlure, C. , Radchuk, V. , Malinowska, A. H. , Curtis, J. M. R. , Naujokaitis‐Lewis, I. , Wintle, B. A. , & Henle, K. (2013). A protocol for better design, application, and communication of population viability analyses. Conservation Biology, 27(4), 644–656. 10.1111/cobi.12076 23692056

[ece39752-bib-0025] R Core Team . (2015). R: A language and environment for statistical computing. R Foundation for Statistical Computing. https://www.R‐project.org/

[ece39752-bib-0026] Reed, J. M. , Mills, L. S. , Dunning, J. B. , Menges, E. S. , McKelvey, K. S. , Frye, R. , Beissinger, S. R. , Anstett, M.‐C. , & Miller, P. (2002). Emerging issues in population viability analysis. Conservation Biology, 16(1), 7–19. 10.1046/j.1523-1739.2002.99419.x 35701959

[ece39752-bib-0027] Santini, L. , Cornulier, T. , Bullock, J. M. , Palmer, S. C. F. , White, S. M. , Hodgson, J. A. , Bocedi, G. , & Travis, J. M. J. (2016). A trait‐based approach for predicting species responses to environmental change from sparse data: How well might terrestrial mammals track climate change? Global Change Biology, 22(7), 2415–2424. 10.1111/gcb.13271 27073017

[ece39752-bib-0028] Shaffer, M. , Watchman, L. H. , Snape, W. J., III , & Latchis, I. K. (2002). Population viability analysis and conservation policy. In Population viability analysis (pp. 123–142). The University of Chicago Press.

[ece39752-bib-0029] Shaffer, M. L. (1981). Minimum population sizes for species conservation. Bioscience, 31(2), 131–134.

[ece39752-bib-0030] Soulé, M. E. (1987). Viable populations for conservation. Cambridge University Press.

