# Supplementary figures and images for: A comparison of population viability measures
Source: Ecol Evol. 2023 Jan 24;13(1):e9752. doi: 10.1002/ece3.9752 (PMC9873871; doi:10.1002/ece3.9752)

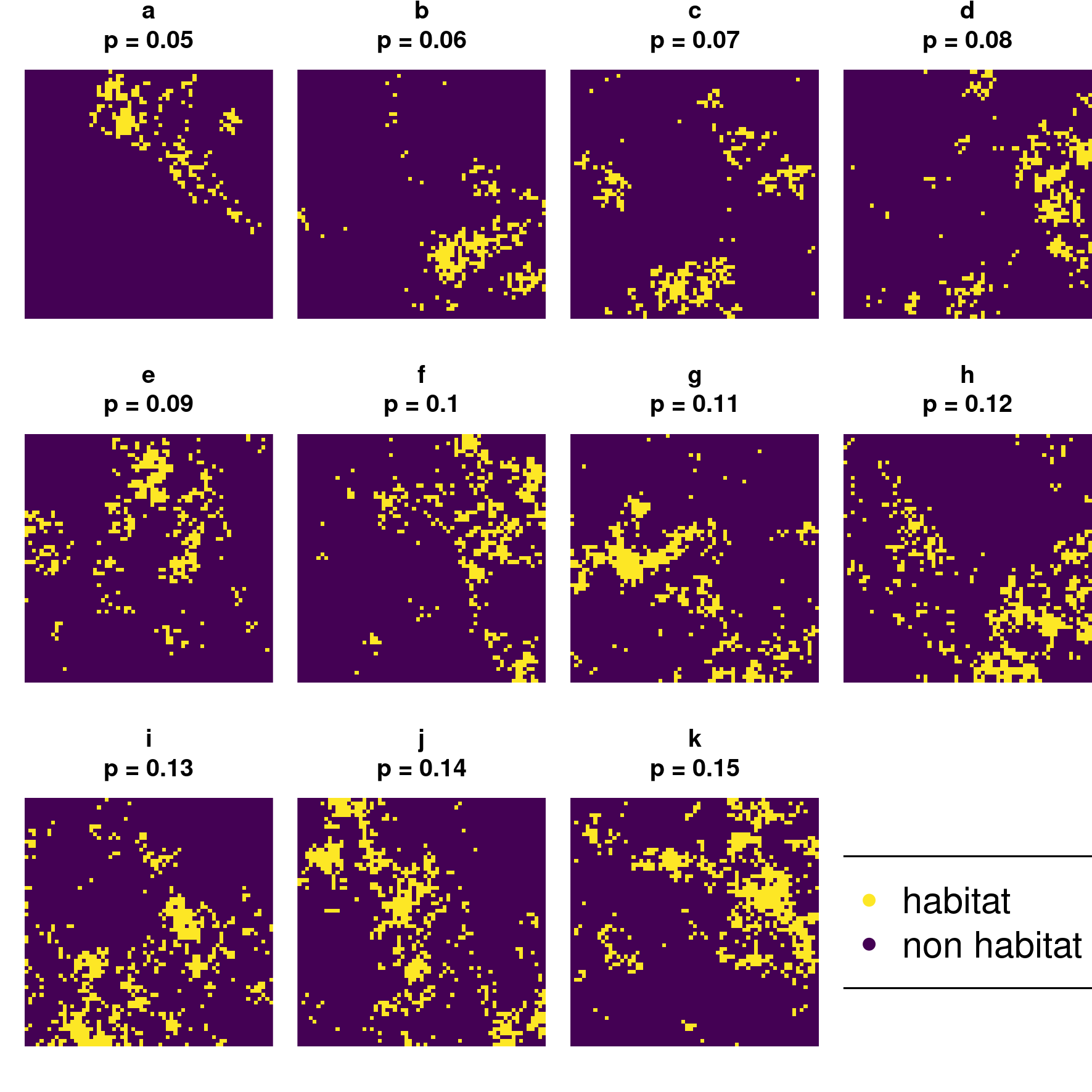

Supplement: Supplementary file 2 — Appendix S2 [file ECE3-13-e9752-s002.png]
